# Flow-cytometric analysis of immune cell populations in patients with depression: relationship with depression severity and electroconvulsive therapy therapeutic outcomes

**DOI:** 10.3389/fncel.2025.1693999

**Published:** 2025-11-20

**Authors:** Karen M. Ryan, Aoife O’Rourke, Christopher Sheridan, Marina Balcells Quintana, Barry Moran, Jean M. Fletcher, Declan M. McLoughlin, Andrew Harkin

**Affiliations:** 1 Trinity College Institute of Neuroscience, Trinity College Dublin, Dublin, Ireland; 2 Department of Psychiatry, School of Medicine, Trinity College Dublin, St. Patrick’s University Hospital, Dublin, Ireland; 3 School of Biochemistry and Immunology, Trinity College Dublin, Trinity Biomedical Sciences Institute, Dublin, Ireland; 4 School of Medicine, Trinity College Dublin, Trinity Biomedical Sciences Institute, Dublin, Ireland; 5 School of Pharmacy and Pharmaceutical Sciences, Trinity College Dublin, Dublin, Ireland

**Keywords:** depression, electroconvulsive therapy, immune, cytokine, flow cytometry

## Abstract

**Introduction:**

Immunological changes are implicated in the pathophysiology of depression. We aimed to assess phenotype and frequency of immune cell subtypes, including an assessment of regulatory T cells and production of cytokines by T cell subsets following stimulation.

**Methods:**

Using a flow cytometric analysis, peripheral blood samples obtained from medicated patients with depression (*n* = 20) were analysed and compared to age-and sex-matched healthy controls (*n* = 21), and in patients with depression after electroconvulsive therapy (ECT) in a real-world clinical setting. Depression severity was assessed using the Hamilton Depression Rating Scale (HAM-D24).

**Results:**

A reduction in the frequencies of CD19+ B cells and IL-17+ CD8 T cells was evident in depressed patients compared to healthy controls. For a subgroup of depressed patients assessed pre- versus post-ECT, there was no change in phenotype, frequency or function of immune cell subtypes within 72 hours of completing treatment. Further exploratory analyses found that baseline CD16-CD14+ classical monocyte frequency correlated with change in HAM-D24 score post-ECT, indicating that a higher frequency of classical monocytes at baseline is associated with greater symptom improvement after treatment. A reduced number of CCR7-CD45RO+ effector memory T cells was also found to be associated with an improvement in symptoms post-ECT.

**Discussion:**

Overall, these results demonstrate that flow cytometry is useful for immune profiling to identify altered adaptive immune features in depression and potential biomarkers of ECT response. In particular, changes in classical monocytes and effector memory T cells were associated with treatment response in patients with unipolar depression.

## 1 Introduction

A growing body of research indicates that major depressive disorder (MDD) is accompanied by measurable shifts in immune cell populations and inflammatory signaling. Peripheral blood analyses consistently confirm increased numbers of both innate and adaptive immune cells in cohorts of patients with MDD. A comprehensive meta-analysis of 27 studies reported increases in absolute counts of white blood cells: granulocytes, neutrophils, monocytes, CD4^+^ T cells, natural killer (NK) cells, CD19^+^ B cells, and activated T cells (particularly CD25^+^ and HLA-DR^+^ subsets) in MDD patients compared with healthy controls, with relative reductions in helper T (Th)1 and Th2 cells but largely unchanged numbers of other T cell subsets, including CD3^+^, CD8^+^, naive and memory T cells and regulatory T (Treg) cells (Foley et al., 2023). Reduced Th17 cells and imbalanced Th1/Th2 ratios further suggest dysregulated adaptive immunity. Overall, however, immunophenotyping studies in depression have demonstrated no unequivocal pattern but rather moderate and context-dependent changes (Poletti et al., 2024). A subset of depressed patients (∼ 30%) consistently exhibit chronic, low-grade inflammation, characterized by expanded myeloid/lymphoid cell populations and elevated inflammatory markers like C-reactive protein (CRP) and IL-6 (Osimo et al., 2020; see also Supplementary Table 1).

Antidepressant treatments have been associated with increased CD4 and CD8 T cell counts and NK cell activity, while simultaneously reducing humoral immune markers of inflammation like CRP and IL-2 (Goyal et al., 2017). Selective serotonin reuptake inhibitors (SSRI) produce modulatory effects on immune cell populations in patients with depression. While short-term SSRI use may transiently reduce NK cell activity (Hannestad et al., 2011), long-term SSRI treatment (e.g., 52 weeks) increased NK cell counts and B cell populations (Hernández et al., 2010). SSRI-enhanced B cell proliferation and Treg cell activity (Hernández et al., 2010; Köhler-Forsberg et al., 2019). Additionally, SSRIs are reported to reduce production of inflammatory cytokines including IL-6, which correlated with improved depressive symptoms (Strawbridge et al., 2015).

Electroconvulsive therapy (ECT) is the most acutely effective antidepressant treatment available for severe, often treatment-resistant, depression (Kirov et al., 2021, Mutz et al., 2019), yet its mechanism of action remains unknown. Accumulating evidence suggests that ECT has immunomodulatory properties (Dellink et al., 2025; Ryan and McLoughlin, 2019; van Buel et al., 2015). In this regard, ECT has been associated with a transient increase in levels of pro-inflammatory cytokines such as IL-6 and TNF (Fluitman et al., 2011; Lehtimaki et al., 2008). A positive association between baseline (pre-ECT) TNF mRNA and change in 24-item Hamilton Depression Rating Scale (HAM-D24) score post-ECT has been reported in unipolar depressed patients (Ryan et al., 2022). ECT further produces changes in cellular immune parameters including elevated absolute numbers of granulocytes, monocytes, and NK cells (Fluitman et al., 2011), an acute reduction in absolute T cell numbers (Fluitman et al., 2011), and increase in the percentage and absolute numbers of active T cells (Moschny et al., 2020).

**FIGURE 1:**
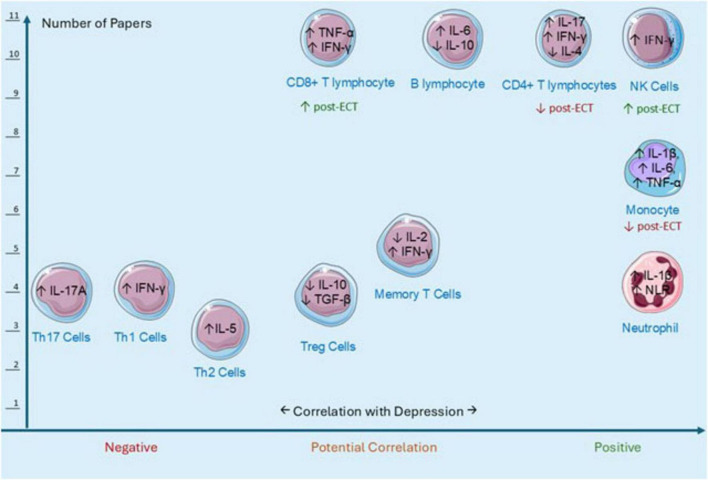
Immune cell populations and soluble immune markers associated with MDD.

A summary of immune cell populations and soluble immune markers associated with MDD is further illustrated in Figure 1. Effects of ECT are shown within each cell type. Further details of the effects of ECT on immune cell types and markers, and their association with remission and recovery from MDD are provided in Supplementary Table 2. There is growing evidence that immune cell alterations reflect the underlying pathophysiology of depression, which may help to differentiate subtypes of the disorder, and predict treatment effects and clinical outcomes (Gentile et al., 2021; Kamimura et al., 2020; Wrona, 2006).

Flow cytometry is indispensable for high-resolution assessment of immune cell subsets surpassing traditional differential leukocyte counts. It is particularly well-suited for immunophenotyping, allowing detailed analysis of immune cell subsets, activation states and functional markers in peripheral blood samples (Perfetto et al., 2004). Adaptive immune markers including CD3, CD4, CD8, and Treg cell markers (CD25, CD127) enable identification of T cell subset imbalances, while B cell markers (CD19, CD20) and memory/naïve T cell discrimination (CD45RA, CCR7) provide further cellular granularity (Felger and Miller, 2020; Lynall et al., 2020). In the context of depression, flow cytometry is well suited for the screening and characterization of immune cell populations, as research increasingly implicates immune dysfunction affecting both innate and adaptive immunity in the pathogenesis of depressive disorders (Miller and Raison, 2016). Flow cytometry has been utilized to identify alterations in the counts and proportions of various immune cell types, as well as changes in activation states and cytokine production profiles in patients with depression (Foley et al., 2023). It is also widely used to inform immunomodulatory effects of antidepressant treatments that may relate to their therapeutic actions (see Figure 1; Supplementary Tables 1, 2).

To our knowledge, Moschny et al. (2020) is the first of few studies to use flow cytometry to examine immune cell parameters in patients with MDD receiving ECT. Thus, the aims of this exploratory study were: (1) to examine the use of flow cytometry in assessing immune cell subtypes in a cohort of severely clinically depressed patients compared to a group of healthy controls, (2) to determine if there is any relationship between immune cell subtypes and depression severity, and (3) to conduct a preliminary analysis of the immunomodulatory effects of ECT.

## 2 Materials and methods

### 2.1 Participants

Patients with depression were recruited to the first phase of the KEEP-WELL (Ketamine for Depression Relapse Prevention Following ECT; NCT02414932) Trial and underwent ECT for the treatment of a major depressive episode in St. Patrick’s Mental Health Services, Ireland between April 2015 and April 2017, as previously described (Finnegan et al., 2019). Brief-pulse (1.0 ms) ECT was administered twice weekly with hand-held electrodes, with methohexital (0.75 mg/kg–1.0 mg/kg) and succinylcholine (0.5 mg/kg–1.0 mg/kg) used for anesthesia and muscle relaxation, respectively. Patients were maintained on their prescribed medications for the duration of the study.

Patients recruited to the trial met the following inclusion criteria: ≥ 18 years referred for ECT, unipolar major depressive disorder (DSM-IV), 24-item Hamilton Rating Scale for Depression (HAM-D24) (Beckham and Leber, 1985) score ≥ 21, ability to provide valid informed consent. Exclusion criteria were as follows: any condition rendering the patient medically unfit for ECT or general anesthesia, active suicidal intent, dementia, intellectual disability, or Mini Mental State Examination (MMSE) (Folstein et al., 1975) score < 24, lifetime history of bipolar affective disorder, current history of post-traumatic stress disorder, other Axis I diagnosis (DSM-IV), ECT in the 6 months prior to recruitment, alcohol dependence or substance misuse in the 6 months prior to recruitment, pregnancy or breast-feeding, residing in a nursing home, prisoner, patients with a neurological or inflammatory condition, diagnosis of a terminal illness, inability or refusal to provide valid informed consent. Patients taking anti-inflammatory medications were included in the KEEP-WELL trial but excluded from the current analysis.

Healthy controls with no history of mental illness were recruited through local newspaper and social media advertisements. Demographic and clinical characteristics of study participant s are provided in Table 1. A subset of 20 patients from the Keep Well trial, not taking anti-inflammatory medications, were included in exploratory analyses. Of these 20 patients, a reduced set of 12 patients, from whom samples were available pre and post ECT, were included for further preliminary analyses. N numbers are provided in Table 1.

**TABLE 1:** Demographic and clinical characteristics of participants.

Variable	Controls (*n* = 21)	Depressed (*n* = 20)	Statistical test
Age, years	57.95 (9.88)	59.65 (12.74)	*t* = 0.48, *p* = 0.64
Sex, No. (%)			
Male	11 (52.38)	11 (55)	χ^2^ = 0.03, *p* = 0.87
Female	10 (47.62)	9 (45)	*t* = 1.05, *p* = 0.30
BMI	26.03 (4.29)	27.51 (4.80)	
Smokers, No. (%)	6 (28.57)	4 (20)	χ^2^ = 0.41, *p* = 0.52
Education, No. (%)			
Primary	0 (0)	0 (0)	χ^2^ = 5.47, *p* = 0.02
Secondary	6 (28.57)	13 (65)	U < 0.00, *p* = 0.000
Tertiary and quaternary	15 (71.43)	7 (35)	
Baseline/pre-ECT HAM-D24	3.14 (2.50)	29.50 (7.34)	
Follow-up/post-ECT HAM-D24		10.85 (8.63)	
Electrode placement, No. (%)			
Unilateral		16 (80)	
Bitemporal		4 (20)	
Number of ECT sessions		8.70 (3.59)	
Remitters, No. (%)		9 (45)	
Psychotropic medications, No. (%) taking			
SSRI		6 (30)	
Non-SSRI		10 (50)	
Mood stabilizer		10 (50)	
Antipsychotic		13 (65)	
Benzodiazepine		10 (50)	

Data are presented as means with standard deviations (SD) or number (%) per group where appropriate. BMI, body mass index; ECT, electroconvulsive therapy; HAM-D24, Hamilton depression rating scale, 24-item version; SSRI, selective serotonin reuptake inhibitor.

Ethical approval for this study was granted by St Patrick’s University Hospital Research Ethics Committee, and the study was performed in accordance with the Declaration of Helsinki (World Medical Association, 2013). All participants provided written informed consent to participate in this study.

### 2.2 Clinical and demographic information

Clinical and demographic data were documented for both patients with depression and controls. The HAM-D24 was used to assess depression severity and response to ECT. Remission was defined as a ≥ 60% decrease from baseline HAM-D24 and a score ≤ 10 on two consecutive weekly ratings.

### 2.3 Peripheral blood collection and processing

Fasting blood samples were taken from patients with depression in the morning before the first ECT treatment and at 1–3 days post-treatment. Fasting control blood samples were taken in the morning on assessment days. After the participant had rested quietly for 45 min, peripheral blood was collected into a sodium heparin Ficoll Cell Preparation Tube (CPT; BD, UK). Following transport of the CPT tube from the hospital site to the laboratory [within 2 h (h)], peripheral blood mononuclear cells (PBMC) were harvested per manufacturer’s instructions. Prior to freezing, the cell pellet was resuspended in 1 ml cell freezing medium [90% fetal bovine serum (FBS)/10% dimethyl sulfoxide (DMSO)] at 4°C. The tubes were stored at –80°C for 24 h in a *Mr Frosty* freezing container and were then transferred to liquid nitrogen for long-term storage.

### 2.4 Flow cytometry

Defrosted cell samples were split in three for analysis. The three fluorochrome panels were as follows: (1) Immune cell panel to define immune cells within the samples; (2) Regulatory T cell panel to characterize Treg cells; (3) Cytokine panel to identify cytokines produced by T cell subsets following stimulation. Panels were optimized to minimize spectral overlap and maximize resolution.

The flow cytometry panels used in this study (Supplementary Table 3) offer a comprehensive and novel approach to characterizing immune alterations in depression and following treatment with ECT. Details of Vendor, panel and codes for each item are included in Supplementary Table 4.

By incorporating markers across a wide range of immune cell types, including T cells, B cells, NK cells, dendritic cells, and myeloid populations, these panels can examine the functionality of both innate and adaptive immunity. This also includes functionally distinct subsets of cells such as Treg cells, Th17 cells, and various monocyte and dendritic cell phenotypes with activation and exhaustion markers (e.g., Ki67, PD-1, CTLA-4), alongside intracellular cytokine staining (e.g., IL-17, IFN-γ, TNF, IL-21), allowing for functional insights on the inflammatory bias within specific cell compartments. Subsets such as CD161^+^ T cells, CD8 T cells and GM-CSF producing T cells are rarely assessed in psychiatric research. This resolution can be used to detect subtle, clinically relevant shifts in immune regulation.

#### 2.4.1 Immune cell panel staining

Single cell suspensions were incubated in complete Iscove’s Modified Dulbecco’s Medium (Sigma-Aldrich, United States) supplemented with 10% FBS, 1% L-glutamine + penicillin + streptomycin (cIMDM) for 5 h. Cells were then washed in phosphate buffered saline (PBS) before staining with 1:1,000 dilution of Fixable Viability Dye (Invitrogen, United States) for 10 min (min) in the dark at room temperature (RT). Cells were incubated with surface binding antibodies for 30 min in the dark at RT. Cells were then washed with PBS and fixed with 1X fixative buffer (FoxP3 Staining Buffer set, Invitrogen) for 20 min in the dark at RT. Cells were then washed and resuspended in PBS. Details of the surface binding antibodies used are provided in Supplementary Table 3.

#### 2.4.2 Regulatory T cell panel staining

Single cell suspensions were incubated in cIMDM at 37°C and 5% CO_2_ for 5 h. Cells were then washed in PBS before staining with a 1:1,000 dilution of the Fixable Viability Dye for 10 min in the dark at RT. Cells were then incubated with surface-binding antibodies for 30 min in the dark at RT. Cells were washed in permeabilization buffer and then incubated with intracellular-labeled antibodies for 30 min in the dark at RT. Cells were subsequently washed in permeabilization buffer and resuspended in PBS. Details of the surface binding and intracellular-labeled antibodies used are provided in Supplementary Table 3.

#### 2.4.3 Cytokine panel staining

Single cell suspensions were incubated in cIMDM with 50 ng/ml phorbol 12-myristate 13-acetate (PMA) and 500 ng/ml ionomycin (Sigma-Aldrich) at 37°C and 5% CO_2_ for 2 h. Then, 5 μg/ml of the Golgi transport blocker brefeldin A was added and cells were incubated for a further 3 h. Cells were then washed in PBS before staining with a 1:1,000 dilution of the Fixable Viability Dye for 10 min in the dark at RT. Cells were incubated with surface-binding antibodies for 30 min in the dark at RT. Cells were then washed in PBS and fixed in 1X fixative buffer for 20 min in the dark at RT. Cells were then washed with permeabilization buffer and incubated with intracellular-labeled antibodies for 30 min in the dark at room temperature. Samples were then washed with permeabilization buffer and resuspended in PBS. Details of the surface binding and intracellular-labeled antibodies used are provided in Supplementary Table 3.

#### 2.4.4 Analysis

All samples were acquired on an Aurora full-spectrum flow cytometer (Cytek Biosciences, Fremont, CA) within 24 h of staining. Flow cytometry analysis was performed using FlowJo software (BD Biosciences, San Jose, CA).

### 2.5 Statistical analysis

All data were analyzed using SPSS version 26 (IBM Corporation, NY, United States). Data were tested for normality using the Shapiro-Wilk test, and data were log transformed prior to analysis where indicated. Statistical analyses were performed for between-group comparisons of demographic and clinical data using independent *t*-tests, Mann-Whitney U tests, or Chi-square tests (X^2^), where appropriate. We adjusted for potential confounders of age, sex and body mass index (BMI; kg/m^2^) using a general linear model for comparison between healthy controls and patients with depression. Correlation analyses were carried out using Pearson’s r or Spearman’s ρ where appropriate to determine relationships between continuous variables. Demographic and clinical data are presented as mean ± standard deviation (SD) or number (%) per group where appropriate. Data are presented as mean ± SD.

## 3 Results

### 3.1 Demographic and clinical information

For this study, we had PBMC samples available from 20 medicated patients with depression recruited to the KEEP-WELL trial and 21 healthy controls, following exclusion of participants with an inflammatory or neurological condition. Samples with poor viability were excluded across individual analyses. Table 1 shows the demographic and clinical characteristics of the participants included in this study.

### 3.2 Flow cytometric analysis of PBMC from healthy controls compared to patients with depression

**FIGURE 2:**
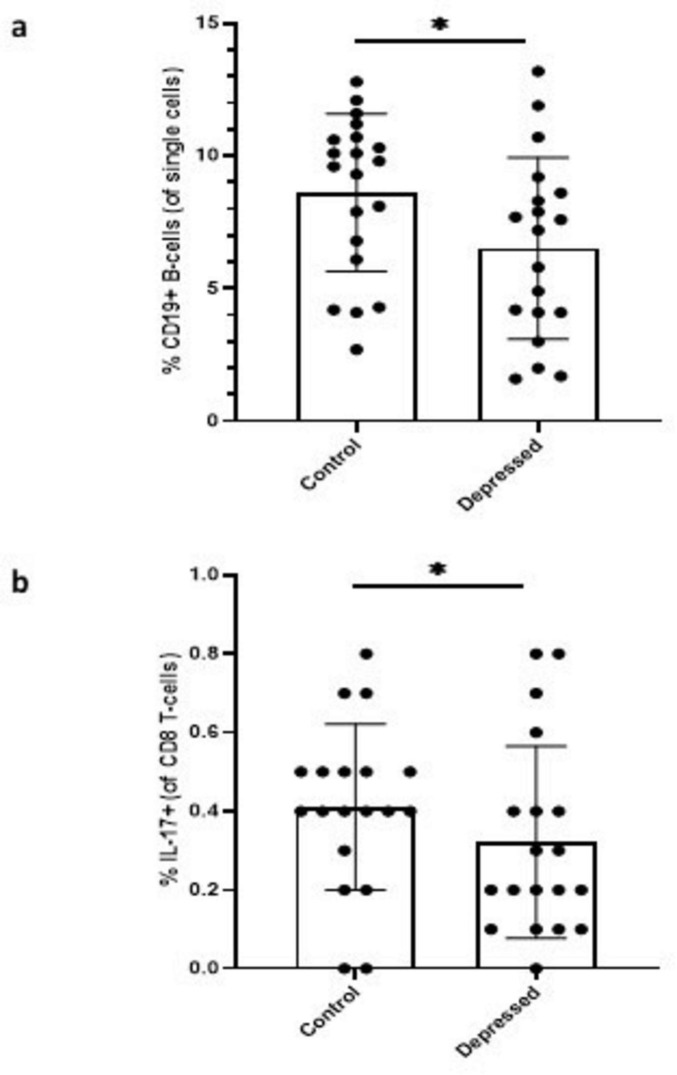
**(a)** % CD19^+^ B-cells (of single cells): The frequency of CD19^+^ B-cells was significantly lower in the depressed patient group when compared to healthy controls. Each dot represents an individual subject. **(b)** % of IL-17^+^ (of CD8 T-cells): The frequency of IL-17^+^ CD8 T-cells was also significantly reduced in the depressed patient group relative to healthy controls. Each dot represents an individual subject. Data are presented as means ± SD. *p < 0.05.

Figure 2a shows that there was a lower % of CD19^+^ B cells in depressed patients when compared to controls (unadjusted analysis: *p* = 0.05) and this difference was statistically significant following adjustment for potential confounders (adjusted analysis: *p* = 0.04). Figure 2b shows that patients with depression had a significantly reduced % of IL-17^+^ CD8 T cells (unadjusted analysis: *p* = 0.02), and this withstood statistical adjustment for potential confounders (adjusted analysis: *p* = 0.04). No other significant differences were noted between groups across any of the three panels (Supplementary Table 5).

### 3.3 Flow cytometric analysis of PBMC from patients with depression pre- and post-treatment with ECT

We conducted an exploratory analysis of flow cytometric changes following ECT in a subset of 12 depressed patients for whom we had samples available pre- and post-ECT. Of these, *n* = 4 were smokers, *n* = 8 were non-smokers, *n* = 9 received treatment with unilateral ECT while *n* = 3 received bilateral ECT, and there were *n* = 5 remitters and *n* = 7 non-remitters. No significant changes were observed in response to ECT across any of the three panels assessed (Supplementary Table 6).

### 3.4 Association between immune cell subtypes and depression severity

**FIGURE 3:**
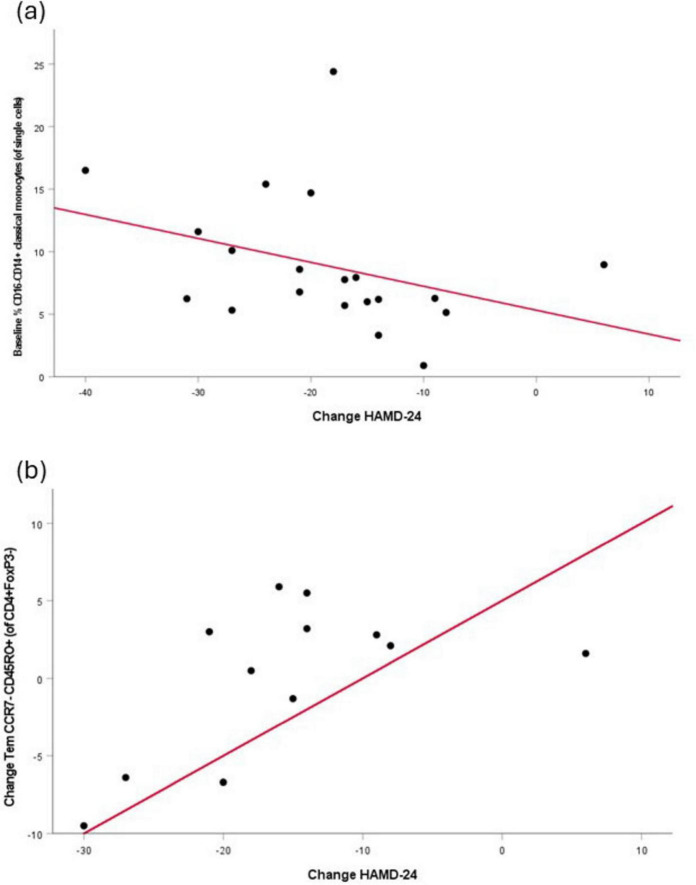
Correlations between immune cell subsets and depressive symptom severity or change. **(a)** % CD16-CD14+ classical monocytes at baseline vs. Change in HAM-D24: Scatter plot showing the association between % CD16-CD14+ classical monocyte cell frequencies at baseline and the change in HAM-D24 scores over the study period. Each dot represents an individual subject; the red line indicates the line of correlation. **(b)** Change in Tem/CCR7^–^CD45RO^+^ cells vs. Change in HAM-D24: Scatter plot depicting the relationship between the change in Tem/CCR7^–^CD45RO^+^ cell frequencies and the change in HAM-D24 scores. Each dot represents an individual subject; the red line indicates the line of correlation. Abbreviations: HAM-D24, Hamilton Depression Rating Scale-24 item; Tem, effector memory T cells.

Correlation analyses were performed to determine the relationship between immune cell subtypes and depression severity, as assessed using the HAM-D24, in the group of patients. A significant moderate negative correlation was noted between baseline CD16-CD14+ classical monocytes and change in HAM-D24 score (Figure 3a; rho = –0.49, *p* = 0.03), indicating that having a higher number of these cells before treatment was associated with greater improvement in depressive symptoms. In addition, a trend toward a significant association between the change in CCR7^–^CD45RO^+^ effector memory T (Tem) cells and the change in HAM-D24 was noted (Figure 3b: *r* = 0.583, *p* = 0.047), suggesting that a reduction in the numbers of these cells may be associated with improved depressive symptoms following ECT.

## Discussion

This is the most comprehensive report to date on phenotype and frequency of immune cell subtypes, that includes an assessment of Treg cells and production of cytokines by T cell subsets following stimulation, as determined using flow cytometric analysis, in PBMC samples obtained from depressed patients and healthy controls and from depressed patients pre- and post-ECT. A reduction in the % CD19^+^ B cells and % IL-17^+^ CD8 T cells was evident in depressed patients compared to healthy controls.

B cell changes have previously been reported in depressed patients where some studies have reported increased total B cell counts (Foley et al., 2023), and others have reported a decrease in the number of these cells when compared to healthy controls (Pavón et al., 2006). A reduction in % CD19^+^ B cells is consistent with a previously reported observation of a decreased frequency of CD19^+^ B cells in a cohort of outpatients with MDD compared to controls (Pavón et al., 2006). More recently, regulatory B cell populations, such as IgD^–^CD27^+^ naïve B cells, CD1d^–^CD5^+^ B cells, and CD24^–^CD38*^hi^* transitional B cells, are reported to be diminished in individuals with severe depression (Ahmetspahic et al., 2018).

IL-17^+^ CD8 T cells, or Tc17 cells, are a pro-inflammatory subset of cytotoxic T lymphocytes that secrete IL-17, a cytokine implicated in both autoimmune and neuroinflammatory processes (do Sacramento et al., 2022). Although this study found a reduction in the percentage of IL-17^+^ CD8^+^ T cells in depressed patients compared to healthy controls, several other studies have reported elevated levels of IL-17^+^ T cells in MDD, both in the CD4^+^ (Th17) and CD8^+^ (Tc17) subsets, linked to greater symptom severity and an increased risk of autoimmune comorbidities (do Sacramento et al., 2022; Alvarez-Mon et al., 2021a). To date data on Tc17 cells and ECT are lacking. In clinical populations, IL-17 levels may be reduced by antidepressant treatment or anti-IL-17 biologics, particularly in individuals with comorbid autoimmune disease (Beurel et al., 2020). Furthermore, experimental models have shown that IL-17 producing T cells can activate microglia and promote neuroinflammation, while neutralizing IL-17 mitigates depressive-like behavior (Beurel and Lowell, 2018).

The results showed no differences in phenotype, frequency or function of immune cell subtypes assessed post-ECT, within 72 h of completing treatment. Moreover, there was no association between the percentage of CD19^+^ B cells or Tc17 cells and mood scores, either when comparing depressed patients to healthy controls or when comparing pre- and post-ECT measurements, refuting the supposition that ECT corrects these changes in conjunction with mood improvement.

Correlation analyses showed a higher number of CD16^–^CD14^+^ classical monocytes before treatment was associated with greater improvement in symptoms. While monocytosis (increase in total monocyte count) is commonly observed in depression, it generally reflects an increase in a less classical, more pro-inflammatory monocyte phenotype as opposed to CD16^–^CD14^+^ cells (Foley et al., 2023; Daray et al., 2024). Several studies report that patients (including medicated patients) with MDD exhibit a decreased proportion and absolute number of classical CD16^–^CD14^+^ monocytes in peripheral blood compared to healthy controls (Nowak et al., 2019; Alvarez-Mon et al., 2021a,b). This reduction is frequently accompanied by an expansion of intermediate and non-classical, pro-inflammatory monocyte subsets. Thus, lower circulating levels of classical monocytes may be associated with a heightened inflammatory profile and greater depression severity.

Higher baseline absolute monocyte counts, largely reflecting classical CD16^–^CD14^+^ monocytes, have been identified as predictors of a better antidepressant response during intravenous ketamine treatment for individuals with treatment-resistant depression. This association remained significant after controlling for age, sex, and BMI, and did not extend to other inflammatory markers such as CRP (Pedraz-Petrozzi et al., 2024).

Elevated levels of classical CD16^–^CD14^+^ monocytes may result in better ECT outcomes as these cells are the principal monocyte subset responsible for anti-inflammatory and tissue-reparative functions (Maffioletti et al., 2021; Pedraz-Petrozzi et al., 2024). Individuals with higher baseline levels of these monocytes are less likely to exhibit persistent, maladaptive inflammatory states that are typically associated with greater depression severity and resistance to therapy (Pedraz-Petrozzi et al., 2024). During ECT, there is an acute activation of the innate immune system, often reflected by transient increases in circulating monocyte counts, where patients with a pre-existing abundance of classical monocytes may undergo a more beneficial immunomodulation, reducing inflammatory dysfunction and promoting recovery (Kirlioglu-Balcioglu et al., 2025; van Buel et al., 2015; Ryan et al., 2022). Furthermore, classical monocytes secrete factors that facilitate neuronal repair and remodeling, providing a cellular environment favorable to neuroplastic changes which contribute to ECT’s antidepressant efficacy (Maffioletti et al., 2021; Pedraz-Petrozzi et al., 2024). Elevated classical monocytes may act not only as participants in the therapeutic cascade triggered by ECT but also as biomarkers of overall immune health, potentially reducing the risk of persistent inflammation that could otherwise affect treatment response (Ryan et al., 2022; Kirlioglu-Balcioglu et al., 2025).

Correlation analyses also showed a reduced number of CCR7^–^CD45RO^+^ Tem cells to be associated with an improvement in symptoms following ECT. CCR7^–^CD45RO^+^ Tem cells lack lymph node-homing receptors and express memory markers that enable rapid effector responses in peripheral tissues (Sallusto et al., 1999). Several studies have reported altered memory and effector T cell profiles in depression, including increased activation of Tem populations (Schiweck et al., 2020; Alvarez-Mon et al., 2021b). These cells are capable of rapid cytokine production and cytotoxic function. While direct evidence of a role for CCR7^–^CD45RO^+^ cells in MDD remains limited, meta-analytic findings indicate that memory T cell subsets are often dysregulated (Schiweck et al., 2020).

Our findings link CD16^–^CD14^+^ classical monocytes with symptom improvement following ECT. Given their abundance, pro-inflammatory responsiveness, and phagocytic efficiency, classical monocytes may actively facilitate recovery. Alterations in CCR7^–^CD45RO^+^ Tem cells were also correlated with change in HAMD score post-ECT potentially reflecting residual immune activation or ongoing low-grade stimulation, though replication is required. It is not unreasonable to suggest that CD16^–^CD14^+^ classical monocytes through cytokine production and antigen production may associated with functional changes in CCR7^–^CD45RO^+^ Tem cells, in a reciprocal interplay. Together, these data point to a coordinated modulation of both innate and adaptive immunity as a potential mechanism underlying ECT’s therapeutic effects.

There are several limitations to this study. First the sample number was small, in our exploratory analysis of depressed patients pre-/post-ECT, and so further studies with larger sample sizes should be conducted to verify the results. Second, all patients were receiving pharmacotherapy during the study and so the effects of medication are unknown. Further studies are required to determine whether the findings are related to depression or medication. Third, we did not adjust for multiple comparisons. As this is an exploratory study strict adjustment for multiples comparisons was not desirable as the data may inform the conduct of subsequent additional dedicated studies to confirm the observed associations (Althouse, 2016). Finally, the use of cryopreserved PBMC in this study is a limitation as the freezing process may have compromised cell viability in certain populations. Consideration should be given to differences in cryopreservation techniques when comparing results of different studies.

Overlap in flow cytometry panels and sample characteristics has led to mixed findings for certain cell types (e.g., lymphocytes, CD8 T cells), underscoring the need for standardized protocols. Functional markers including HLA-DR, CD64, CD69, and PD-1 would offer additional insight into cellular activation and exhaustion states, which are increasingly recognized as relevant to the pathophysiology and prognosis of MDD (Grosse et al., 2016a,b; Strawbridge et al., 2015). The integration of these markers into a standardized panel facilitates the identification of myeloid- vs. lymphoid-biased inflammation and supports longitudinal monitoring in clinical research settings (Lynall et al., 2020). Such panels could also be combined with systemic markers such as CRP.

Research findings to date support the use of flow cytometry as a tool not only for immunophenotyping but also for identifying potential biomarkers and inflammation-related subgroups within depression, thereby informing more targeted and personalized therapeutic strategies (Miller and Raison, 2016; Foley et al., 2023). Köhler-Forsberg et al. (2019) reported a meta-analysis of clinical trials supporting the efficacy of anti-inflammatory treatment on major depressive disorder. In other studies, chronic, low-grade inflammation may serve as a potential predictive biomarker for treatment selection. Immunological heterogeneity in depression subtypes with markers like neutrophil to lymphocyte ratio (NLR) which is normal/low in melancholic vs. elevated in atypical, CD4:CD8 T cell ratios which may be preserved vs. reduced, and cortisol profiles which may be elevated vs. normal, highlights the potential for subtype-specific treatment, integrating immune profiling into clinical decision-making (Lamers et al., 2013; Drevets et al., 2022; Ryan et al., 2022).

With continued research in this area, use of flow cytometric methods may enable stratification of patients into immune-based subtypes (e.g., myeloid- vs. lymphoid-biased inflammation), which correlate with symptom severity and treatment response. By linking immune cell changes (e.g., monocytosis) to cytokine dysregulation (IL-6, TNF), flow cytometry provides further insights into immunoregulatory functions, reinforcing its clinical and research utility. A hypothetical panel for elucidating further immune changes induced by ECT is proposed in Table 2. An understanding of how the immune system is regulated in central and peripheral compartments and the relationship of changes to neuronal function within limbic brain circuits is also required. Immune markers may be of translational value toward biomarker-informed personalized treatment or development of targeted antidepressants with similar efficacy as ECT.

**TABLE 2:** A hypothetical panel of markers for flow cytometric immune-profiling of MDD.

Cell type	Markers	Relevance to MDD	References
Classical monocytes	CD14 high CD16 low	Altered in MDD, especially in inflammation-associated subtypes	(Moschny et al., 2020; Yrondi et al., 2018; Du et al., 2024)
Non-classical monocytes	CD14 low CD16 high	Altered in MDD, especially in inflammation-associated subtypes	(Moschny et al., 2020; Yrondi et al., 2018; Du et al., 2024)
NK Cells	CD56, CD16	Subset alterations observed in MDD	(Atanackovic et al., 2004; Euteneuer et al., 2014; Hernandez et al., 2010; Maes et al., 1992; Ravindran et al., 1996, 1999; Rothermundt et al., 2001)
Granulocytes	CD15, CD16	Altered in inflammation-associated MDD	(Moschny et al., 2020; Yrondi et al., 2018; Du et al., 2024)
T Cells (general)	CD3, CD4, CD8	Imbalance in subsets linked to MDD and treatment response	(Felger and Miller, 2020; Lynall et al., 2020)
Regulatory T Cells	CD25, CD127	Regulatory imbalance associated with immune dysregulation in MDD	(Becking et al., 2018; Ghosh et al., 2020; Grosse et al., 2016a,b)
B Cells	CD19, CD20	Used to assess adaptive immune alterations in MDD	(Atanackovic et al., 2004; Euteneuer et al., 2014; Lynall et al., 2020; Maes et al., 1992; Pavón et al., 2006; Ravindran et al., 1996, 1999)
Memory/Naïve T cells	CD45RA, CCR7	Discriminate T cell memory status; linked to disease progression and treatment response	(Euteneuer et al., 2014; Maes et al., 1992)

Graphical representation of immune cell population changes associated with major depressive disorder (MDD), adapted from Foley et al. (2023). The *Y*-axis represents the number of studies examining each immune cell type in the context of depression. The *X*-axis reflects the strength and direction of the association between immune cell abundance and depression severity, based on standardized mean differences (SMD), 95% confidence intervals (CI), and *p*-values reported in Foley et al. (2023). Cell-specific alterations in cytokine production observed in depression, as well as reported modulatory effects of electroconvulsive therapy (ECT), are illustrated within each cell type. A summary table of the figure, including quantitative data and references, is provided in Table 1.

Recent advances in immunophenotyping have highlighted the potential of flow cytometry panels to stratify immune alterations in MDD. Key populations implicated include classical and non-classical monocytes (CD14, CD16), NK cell subsets (CD56, CD16), and granulocytes (CD15, CD16), all of which have been shown to be altered in individuals with depression, particularly in inflammation-associated subtypes (Miller and Raison, 2016).

## Data Availability

The raw data supporting the conclusions of this article will be made available by the authors, without undue reservation.
