# Supplementary material for: Flow-cytometric analysis of immune cell populations in patients with depression: relationship with depression severity and electroconvulsive therapy therapeutic outcomes
Source: Front Cell Neurosci. 2025 Nov 20;19:1693999. doi: 10.3389/fncel.2025.1693999 (PMC12676710; doi:10.3389/fncel.2025.1693999)
Supplement: Supplementary file 1 [file Table_1.docx]

Supplementary Table 1: Changes in immune cell populations and soluble immune markers associated with MDD compared to healthy controls.

| **Cell Subset** | **Change in Depression** | **Cytokine expression/ production** | **References** |
| --- | --- | --- | --- |
| CD4+ T Cells | Increased, some populations decreased | ↑ IL-17, ↑ IFN-γ, ↑ IL-4 | Alvarez-Mon et al., 2021a,b;   Daria et al., 2020; Lu et al., 2023 |
| NK Cells | Subset populations increased,  functional impairment | ↑ IFN-γ | Foley et al., 2023 |
| B cells | Regulatory B cell subsets reduced | ↑ IL-6, ↑ IL-10 | Foley et al., 2023; Glass et al., 2022;   Anjum et al., 2020 |
| Monocytes | Increased | ↑ IL-1β, ↑ IL-6, ↑ TNF-α | Suarez et al., 2003;   Roohi et al., 2021 |
| Neutrophils | Increased, reduced functionality, increased NLR | ↑ IL-1β, ↑ NLR | Ghafori et al., 2024;  Kong et al., 2021; Farooq et al., 2017 |
| CD8+ T Cells | Increased activation | ↑ TNF-α | Rachayon et al., 2024;  Jiao et al., 2025 |
| Total Lymphocytes | Altered total count | Not determined | Foley et al., 2023 |
| Memory T Cells | Increased, premature aging, loss of priming | ↑ IL-2, ↑ IFN-γ | Schiweck et al., 2020;   Suhee et al., 2023 |
| Treg | Reduced expansion | ↓ IL-10, ↓ TGF-β | Jovanovic et al., 2021;   Alvarez-Mon et al., 2021a,b; Musil et al., 2011 |
| Th17 | Altered cytokine expression | ↑ IL-17A | Beurel et al., 2018 |
| Th1 | Altered cytokine expression | ↑ IFN-γ | Myint et al., 2005 |
| Th2 | Moderate reduction,  altered cytokine expression | ↑ IL-5 | Moulton et al., 2025 |

Changes in immune cell populations and soluble immune markers associated with MDD compared to healthy controls. NK cells: Natural Killer cells; NLR: Neutrophil: Lymphocyte ratio

Supplementary Table 2: Immunomodulatory effects of ECT in depression and association with treatment outcomes

| **Cell Type/Marker** | **Effect of ECT** | **Correlation**  **with Remission/Recovery** | **Reference** |
| --- | --- | --- | --- |
| Monocytes | Increased post-ECT | Risk factor; monocytes linked to symptom improvement in some ECT studies | Moschny et al., 2020; Yrondi et al., 2018; Du et al., 2024. |
| CD4+ T cells (total) | Decreased post-ECT | Higher naive CD4+ linked to remission; mixed for total CD4+ | Moschny et al., 2020; Yrondi et al., 2018; Du et al., 2024. |
| CD8+ T cells (total) | Increased post-ECT | Not specifically linked to remission | Moschny et al., 2020; Yrondi et al., 2018; Du et al., 2024. |
| NK cells | Increased post-ECT; linked to immune shifts | Post-ECT ↑ NK may support recovery (immune modulation) | Moschny et al., 2020; Yrondi et al., 2018; Du et al., 2024. |
| Pro-inflammatory cytokines (TNF-α, IL-6) | Decreased after multiple sessions | May reflect symptom relief (less inflammation) | Goldfarb et al., 2020; Rush et al., 2016; Dellink et al., 2025 |
| Kynurenine Pathway Activation Markers | Increased post-ECT | Increase during treatment + post-ECT correlates with symptom improvement | Dellink et al., 2025;  Ryan et al., 2024 |

Supplementary Table 3: Cytometric fluorochrome staining panels for phenotype, frequency and functional analysis of PBMCs

| Immune Cell Panel | | | | | Cytokine Panel | | | | Regulatory T cells Panel | | | |
| --- | --- | --- | --- | --- | --- | --- | --- | --- | --- | --- | --- | --- |
| Location | Target | Fluorochrome | | µl Ab / 50 µl | Location | Target | Fluorochrome | µl Ab / 50 µl | Location | Target | Fluorochrome | µl Ab / 50 µl |
| Extracellular | FixVia | eF506 | 0.05 | | Extracellular | FixVia | eF506 | 0.05 | Extracellular | FixVia | eF506 | 0.05 |
|  | CD3 | BV711 | 1.25 | |  | CD3 | BV711 | 1.25 |  | CD3 | BV711 | 1.25 |
|  | CD4 | PE-CF594 | 1.25 | |  | CD4 | APC-R700 | 2.5 |  | CD4 | PE-CF594 | 1.25 |
|  | CD8 | PE-Cy7 | 1.25 | |  | CD8 | APC-eF780 | 1.25 |  | CD25 | PE-Cy7 | 2.5 |
|  | ɣδ TCR | BV650 | 2.5 | |  | ɣδ TCR | BV650 | 2.5 |  | CD39 | PerCP-eF710 | 2.5 |
|  | CD11c | BV480 | 1.25 | |  | CD161 | PE-Cy5 | 5 |  | CD45RO | BV421 | 2.5 |
|  | CD14 | BV786 | 1.25 | | Intracellular | IL-2 | PE-CF594 | 1.25 |  | CD49b | A647 | 1.25 |
|  | CD16 | PerCP-Cy5.5 | 1.25 | |  | IL-4 | BV421 | 1.25 |  | CD127 | APC-eF780 | 2.5 |
|  | CD19 | APC-R700 | 1.25 | |  | IL-17 | AF488 | 1.25 |  | CD161 | PE-Cy5 | 5 |
|  | CD49b | A647 | 1.25 | |  | IL-21 | A647 | 5 |  | CCR7 | APC | 2.5 |
|  | CD56 | BV570 | 1.25 | |  | IL-22 | PE-Cy7 | 1.25 |  | PD-1 | BV650 | 2.5 |
|  | CD64 | BV421 | 1.25 | |  | TNF | PerCP-Cy5.5 | 1.25 |  | CTLA-4 | BV786 | 1.25 |
|  | CD123 | BV605 | 1.25 | |  | IFN-ɣ | BV605 | 0.625 | Intra-cellular | FoxP3 | PE | 1.25 |
|  | CD127 | APC-eF780 | 2.5 | |  | GM-CSF | PE | 1.25 |  | Ki67 | AF488 | 1.25 |
|  | CD161 | PE-Cy5 | 5 | |  |  |  |  |  |  |  |  |
|  | HLA-DR | PE | 1.25 | |  |  |  |  |  |  |  |  |
|  | LAG-3 | FITC | 2.25 | |  |  |  |  |  |  |  |  |

| Supplementary Table 3: Flow Cytometric Analysis of PBMCs from Healthy Controls vs. Patients with Depression Pre-ECT |
| --- |
|  |

Supplementary Table 4: Vendor, panel and codes for flow cytometry materials

Supplementary Table 4: Vendor, panel and codes for flow cytometric items

| Supplementary Table 5: Flow Cytometric Analysis of PBMCs from Healthy Controls vs. Patients with Depression Pre-ECT | | | | | |
| --- | --- | --- | --- | --- | --- |
| **Immune Cell Panel** | | | | | |
|  | **Control** | **Depressed Pre-ECT** | **Unadjusted p-value** | **Adjusted p-value ^c^** | **Cohen’s d Effect Size** |
| **T cells** | | | | | |
| % CD3+ T cells (of single cells) | 65.83 ± 9.81 | 66.79 ± 10.01 | 0.76 | 0.69 | 0.10 |
| % Lag3^-^ T cells (of CD3+ T cells) ^a^ | 99.18 ± 0.91 | 99.03 ± 0.92 | 0.38 |  | 0.16 |
| % CD4^+^ T cells (of CD3+ T cells) ^a^ | 63.65 ± 14.64 | 64.53 ± 11.67 | 0.72 |  | 0.07 |
| % CD161^+^ CD4^+^ T cells (of CD4+ T cells) | 10.39 ± 4.06 | 11.37 ± 5.08 | 0.50 | 0.63 | 0.21 |
| % CD8^+^ T cells (of CD3+ T cells) ^b^ | 27.97 ± 12.02 | 27.92 ± 10.08 | 0.95 | 0.89 | 0 |
| % CD161^+^ CD8 T cells (of CD8 T cells) ^b^ | 10.78 ± 4.41 | 13.10 ± 8.70 | 0.58 | 0.74 | 0.34 |
| % CD161^bright^ CD8 T cells (of CD8 T cells) ^b^ | 6.35 ± 3.65 | 5.98 ± 5.83 | 0.28 | 0.43 | 0.08 |
| **B cells** | | | | | |
| % CD19^+^ B cells (of single cells) | 8.62 ± 2.97 | 6.56 ± 3.34 | 0.05 | **0.04** | 0.66 |
| % HLA-DR^+^CD49b^-^ (of B cells) ^a^ | 92.91 ± 2.79 | 91.90 ± 3.34 | 0.25 |  | 0.33 |
| % HLA-DR^+^CD49^+^ (of B cells) | 4.40 ± 1.66 | 4.03 ± 2.26 | 0.56 | 0.66 | 0.19 |
| **Dendritic Cells** | | | | | |
| % CD11c^+^ ^b^ (of single cells) | 3.93 ± 1.68 | 3.40 ± 2.04 | 0.96 | 0.91 | 0.28 |
| % CD11c^-^CD123^+^ ^a^ (of single cells) | 0.44 ± 0.25 | 0.43 ± 0.36 | 0.43 |  | 0.03 |
| **Natural Killer Cells** | | | | | |
| % CD56^hi^CD16^lo a^ (of single cells) | 0.71 ± 0.42 | 0.58 ± 0.37 | 0.26 |  | 0.33 |
| % CD56^lo^CD16^hi^ (of single cells) ^b^ | 5.79 ± 5.04 | 5.73 ± 5.20 | 0.87 | 0.77 | 0.01 |
| **Monocytes** | | | | | |
| % CD64^+^  (of single cells) ^a^ | 8.29 ± 4.98 | 9.52 ± 6.21 | 0.85 |  | 0.22 |
| % CD16^+^CD14^+^ intermediate monocytes (of single cells) ^b^ | 0.11 ± 0.12 | 0.13 ± 0.12 | 0.32 | 0.22 | 0.17 |
| % CD16^-^CD14^+^ classical monocytes (of single cells)  ^a^ | 7.61 ± 4.58 | 8.89 ± 5.39 | 0.74 |  | 0.26 |
| % CD16^+^CD14^-^ non-classical monocytes (of single cells)  ^b^ | 0.01 ± 0.02 | 0.01 ± 0.01 | 0.88 | 0.90 | 0 |
|  |  |  |  |  |  |
| **Regulatory T Cells Panel** | | | | | |
|  | **Control** | **Depressed pre-ECT** | **Unadjusted p-value** | **Adjusted p-value ^c^** | **Cohen’s d Effect Size** |
| **Treg cells (FoxP3^+^CD25^+^CD127^lo^CD4^+^CD3^+^)** | | | | | |
| % Treg cells (of CD4^+^ T cells) **^a^** | 5.80 ± 1.83 | 5.18 ± 1.22 | 0.51 |  | 0.40 |
| % Ki67^+^ **^b^** (of Treg cells) | 7.55 ± 2.42 | 7.86 ± 4.29 | 0.78 | 0.66 | 0.09 |
| % CD39^+^ (of Treg cells) | 30.52 ± 11.99 | 35.05 ± 16.38 | 0.32 | 0.36 | 0.32 |
| % CD49b^+^ (of Treg cells) | 2.71 ± 1.77 | 2.54 ± 1.28 | 0.71 | 0.86 | 0.11 |
| % PD-1^+^ (of Treg cells) **^b^** | 2.17 ± 3.28 | 1.83 ± 2.13 | 0.99 | 0.94 | 0.12 |
| % CTLA-4^+^ (of Treg cells) **^b^** | 5.12 ± 4.98 | 3.99 ± 3.54 | 0.49 | 0.53 | 0.26 |
| %Naïve CCR7^+^CD45RO^-^ (of Treg cells) | 31.99 ± 14.11 | 31.18 ± 8.00 | 1.00 |  | 0.07 |
| % Tcm CCR7^+^CD45RO^+^ (of Treg cells) | 19.92 ± 5.78 | 17.33 ± 6.96 | 0.28 | 0.24 | 0.40 |
| % Tem CCR7^-^CD45RO^+^ (of Treg cells) | 43.84 ± 11.34 | 47.47 ± 9.90 | 0.36 | 0.26 | 0.34 |
| Ratio Th17:Tregs | 0.22 ± 0.15 | 0.29 ± 0.15 | 0.13 | 0.21 | 0.47 |
| **Non-Treg cells (FoxP3^-^CD4^+^)** | | | | | |
| %Tem CCR7^-^CD45RO^+^ (of CD4+FoxP3-) **^a^** | 22.36 ± 14.16 | 21.89 ± 9.20 | 0.54 |  | 0.04 |
| %Tcm CCR7^+^CD45RO^+^ (of CD4+FoxP3-) | 18.96 ± 10.72 | 15.83 ± 8.09 | 0.36 | 0.22 | 0.33 |
| %PD1^+^ (of CD4+FoxP3-) **^b^** | 1.77 ± 2.91 | 1.41 ± 1.93 | 0.96 | 0.86 | 0.15 |
| % Naïve CCR7^+^CD45RO^-^ (of CD4+FoxP3-) | 51.57 ± 17.80 | 58.80 ± 12.72 | 0.35 | 0.22 | 0.47 |
| % Ki67^+^  (of CD4+FoxP3-) | 1.25 ± 0.51 | 1.22 ± 0.52 | 0.86 | 0.90 | 0.06 |
| % CTLA4^+^ (of CD4+FoxP3-) **^a^** | 2.09 ± 1.63 | 1.65 ± 1.09 | 0.55 |  | 0.32 |
| % CD39^+^ (of CD4+FoxP3-) | 2.36 ± 1.15 | 2.03 ± 1.40 | 0.41 | 0.36 | 0.26 |
| % CD49b^+^ (of CD4+FoxP3-) **^b^** | 1.04 ± 0.88 | 0.91 ± 0.68 | 0.44 | 0.51 | 0.17 |
| **Cytokine Panel** | | | | | |
|  | **Control** | **Depressed pre-ECT** | **Unadjusted p-value** | **Adjusted p-value ^c^** | **Cohen’s d Effect Size** |
| **CD8 T Cells** | | | | | |
| % IL-17^+^ (of CD8+ T cells) ^b^ | 0.41 ± 0.21 | 0.32 ± 0.24 | **0.02** | **0.04** | 0.40 |
| % IFNy^+^ (of CD8+ T cells) | 44.44 ± 15.74 | 41.52 ± 18.76 | 0.61 | 0.43 | 0.17 |
| % TNF^+^ (of CD8+ T cells) **^a^** | 19.78 ± 15.26 | 24.53 ± 14.85 | 0.25 |  | 0.32 |
| % IL-2^+^ (of CD8+ T cells) | 18.82 ± 6.84 | 25.54 ± 12.45 | 0.05 | 0.08 | 0.67 |
| % IL-4^+^ (of CD8+ T cells) **^b^** | 2.26 ± 2.30 | 3.84 ± 4.14 | 0.17 | 0.21 | 0.47 |
| % IL-22^+^ (of CD8+ T cells) **^b^** | 0.45 ± 0.32 | 0.76 ± 0 .87 | 0.46 | 0.53 | 0.47 |
| % IL-21^+^ (of CD8+ T cells) ) **^a^** | 6.33 ± 7.61 | 6.04 ± 7.75 | 0.60 |  | 0.04 |
| % GM-CSF^+^(of CD8+ T cells) **^b^** | 5.91 ± 2.92 | 8.86 ± 5.63 | 0.09 | 0.11 | 0.66 |
| **CD4 T Cells** | | | | | |
| % IL-17^+^ (of CD4 T cells) | 1.37 ± 0.76 | 1.71 ± 0.92 | 0.23 | 0.30 | 0.40 |
| % IFN-γ^+^ (of CD4 T cells) **^b^** | 17.81 ± 10.31 | 16.94 ± 7.85 | 0.93 | 0.97 | 0.09 |
| % TNF^+^ (of CD4 T cells) | 27.75 ± 14.90 | 26.88 ± 7.86 | 0.82 | 0.77 | 0.07 |
| % IL-2^+^ (of CD4 T cells) | 38.52 ± 11.02 | 41.51 ± 8.98 | 0.37 | 0.59 | 0.30 |
| % IL-4^+^ (of CD4 T cells) **^a^** | 2.37 ± 1.99 | 1.88 ± 1.07 | 0.78 |  | 0.31 |
| % IL-21^+^ (of CD4 T cells) **^a^** | 3.64 ± 5.29 | 2.65 ± 3.01 | 0.37 |  | 0.23 |
| % IL-22^+^ (of CD4 T cells) **^b^** | 1.15 ± 0.56 | 1.59 ± 1.51 | 0.28 | 0.38 | 0.39 |
| % GM-CSF^+^ (of CD4 T cells) **^b^** | 6.94 ± 4.26 | 6.34 ± 3.33 | 0.91 | 0.95 | 0.16 |

Data are presented as means ± SD. For % GM-CSF+ (of CD8+ T cells), N = 11 cases.

^a^ Data were not normally distributed following log_10_ transformation, therefore a Mann-Whitney U test was performed.

^b^ Log_10_ transformed data were used for the statistical analysis.

^c^ Adjusted for age, sex, BMI.

| Supplementary Table 6: Flow Cytometric Analysis of PBMCs from Patients with Depression Pre- and Post-ECT | | | | |
| --- | --- | --- | --- | --- |
| **Immune Cell Panel** | | | | |
|  | **Depressed Pre-ECT** | **Depressed Post-ECT** | **p-value** | **Cohen’s d Effect Size** |
| **T-cells** | | | | |
| % CD3+ T-cells ^a^ (of single cells) | 66.47 ± 11.47 | 64.69 ± 10.41 | 0.64 | 0.16 |
| % Lag3^-^ T cells **^a^** (of CD3+ T cells) | 98.74 ± 1.10 | 99.00 ± 1.07 | 0.70 | 0.24 |
| % CD4^+^ T cells (of CD3+ T cells) | 65.48 ± 7.57 | 63.00 ± 10.68 | 0.12 | 0.27 |
| % CD161^+^ T cells ^a^ (of CD4+ T cells) | 9.30 ± 3.54 | 10.52 ± 5.12 | 0.70 | 0.28 |
| % CD8^+^ % T cells (of CD3+ T cells) | 27.11 ± 7.71 | 29.38 ± 9.28 | 0.06 | 0.27 |
| % CD161^+^ (of CD8 T cells) **^a^** | 11.63 ± 6.65 | 13.97 ± 12.57 | 0.35 | 0.23 |
| % CD161^+^ CD161^bright^ (of CD8 T cells) ^a^ | 7.10 ± 7.11 | 6.67 ± 5.82 | 0.58 | 0.07 |
| **B-cells** | | | | |
| % CD19+ B cells (of single cells) ^$^ | 6.15 ± 3.40 | 5.82 ± 2.81 | 0.55 | 0.11 |
| % HLA-DR^+^CD49b^-^ (of B cells) | 92.94 ± 1.83 | 92.65 ± 2.96 | 0.78 | 0.12 |
| % HLA-DR^+^CD49^+^ (of B cells) | 3.14 ± 1.47 | 4.04 ± 2.20 | 0.15 | 0.48 |
| **Dendritic Cells** | | | |  |
| % CD11c^+^ (of single cells) | 4.22 ± 2.60 | 4.45 ± 2.76 | 0.51 | 0.09 |
| % CD11c^-^CD123^+^  **^a^** (of single cells) | 0.51 ± 0.45 | 0.38 ± 0.16 | 0.59 | 0.38 |
| **Natural Killer Cells (CD64^-^; CD3^-^ CD19^-^)** | | | | |
| % CD56^hi^CD16^lo^ **^a^** (of single cells) | 0.65 ± 0.41 | 0.70 ± 0.51 | 0.55 | 0.11 |
| % CD56^lo^CD16^hi^ **^a^** (of single cells) | 5.73 ± 6.48 | 6.98 ± 5.03 | 0.18 | 0.22 |
| % CD56^-^CD16^-^ **^a^** (of single cells) | 9.18 ± 4.46 | 9.43 ± 4.59 | 0.75 | 0.06 |
| **Monocytes** | | | | |
| % CD64^+^ **^a^** (of single cells) | 9.20 ± 7.13 | 10.70 ± 5.81 | 0.37 | 0.23 |
| % CD16^+^CD14^+^ intermediate monocytes (of single cells) **^a $^** | 0.10 ± 0.09 | 0.14 ± 0.11 | 0.25 | 0.40 |
| % CD16^-^CD14^+^ classical monocytes (of single cells) **^a $^** | 8.59 ± 6.01 | 9.97 ± 5.74 | 0.48 | 0.23 |
| % CD16^+^CD14^-^ non-classical monocytes (of single cells) **^a $^** | 0.01 ± 0.01 | 0.01 ± 0.02 | 0.58 | 0 |
| % CD64^-^ **^a^** (of single cells) | 15.88 ± 10.35 | 17.43 ± 8.92 | 0.53 | 0.16 |

| Regulatory T Cells Panel | | | | |
| --- | --- | --- | --- | --- |
|  | **Depressed Pre-ECT** | **Depressed Post-ECT** | **p-value** | **Cohen’s d Effect Size** |
| **T-regs (FoxP3+ CD25+; CD127-)** | | | | |
| % Treg cells (of CD4^+^ T cells) | 5.53 ± 1.01 | 5.46 ± 1.69 | 0.92 | 0.05 |
| % CD161^+^ (of Treg cells) | 9.92 ± 3.99 | 7.94 ± 2.77 | **0.03** | 0.54 |
| % Ki67^+^ (of Treg cells) | 6.01 ± 2.43 | 7.43 ± 3.25 | 0.05 | 0.49 |
| % CD39^+^ **^a^** (of Treg cells) | 31.38 ± 13.69 | 31.25 ± 11.89 | 0.94 | 0.01 |
| % CD49b^+^ (of Treg cells) | 2.48 ± 1.31 | 2.09 ± 1.52 | 0.42 | 0.27 |
| % CTLA-4^+^ **^a^** (of Treg cells) | 5.26 ± 3.47 | 6.98 ± 10.96 | 0.72 | 0.21 |
| % Naïve CCR7^+^CD45RO^-^ (of Treg cells) | 32.37 ± 7.04 | 34.11 ± 9.20 | 0.46 | 0.21 |
| % Tcm CCR7^+^CD45RO^+^ (of Treg cells) | 16.08 ± 5.62 | 17.03 ± 6.86 | 0.59 | 0.15 |
| % Tem CCR7^-^CD45RO^+^(of Treg cells) | 47.25 ± 8.27 | 45.38 ± 11.13 | 0.46 | 0.19 |
| Ratio Th17:Tregs | 0.30 ± 0.13 | 0.35 ± 0.21 | 0.25 | 0.27 |
| **Non-T-regs (CD4+ FoxP3-)** | | | | |
| % Tem CCR7^-^CD45RO^+^ **^a^** (of CD4+FoxP3-) | 21.76 ± 10.00 | 21.82 ± 8.78 | 0.75 | 0.01 |
| % Tcm CCR7^+^CD45RO^+^ **^a^** (of CD4+FoxP3-) | 15.23 ± 5.22 | 17.63 ± 8.63 | 0.31 | 0.34 |
| % PD1^+^ **^a^** (of CD4+FoxP3-) | 1.47 ± 2.45 | 1.33 ± 1.81 | 0.92 | 0.06 |
| % Naïve CCR7^+^CD45RO^-^ **^a^** (of CD4+FoxP3-) | 60.93 ± 12.74 | 58.54 ± 15.25 | 0.27 | 0.17 |
| % Ki67^+^ (of CD4+FoxP3-) | 1.09 ± 0.58 | 1.07 ± 0.42 | 0.78 | 0.04 |
| % CTLA4^+^ **^a^** (of CD4+FoxP3-) | 2.22 ± 0.95 | 4.28 ± 8.31 | 0.64 | 0.35 |
| % CD39^+^ (of CD4+FoxP3-) | 1.97 ± 1.34 | 2.30 ± 1.41 | 0.21 | 0.24 |
| % CD49b^+^ **^a^** (of CD4+FoxP3-) | 0.78 ± 0.58 | 0.84 ± 0.87 | 0.53 | 0.08 |

Data are expressed as mean ± SD. Paired t-test. n = 12. ^$^ n = 11

**^a^** Data were not normally distributed so were analysed using a Wilcoxon Signed Rank test.
